# Comparative Study of Damping on Pultruded GFRP and Steel Beams

**DOI:** 10.3390/polym13132201

**Published:** 2021-07-02

**Authors:** Vitor Dacol, Elsa Caetano, João Ramoa Correia

**Affiliations:** 1 CONSTRUCT (ViBEST), Faculty of Engineering (FEUP), Universidade do Porto, 4200-465 Porto, Portugal; ecaetano@fe.up.pt; 2 CERIS, DECivil, IST, Universidade de Lisboa, 1049-001 Lisbon, Portugal; joao.ramoa.correia@tecnico.ulisboa.pt

**Keywords:** composites, GFRP, steel, viscoelasticity, natural frequency, damping, dynamic behaviour, footbridge vibrations

## Abstract

The use of glass fibre reinforced polymer (GFRP) composites in civil engineering structures has seen considerable growth in recent years due to their high strength, low self-weight, and corrosion resistance, namely when compared to traditional materials, such as steel and reinforced concrete. To enable the structural use of GFRP composite materials in civil engineering applications, especially in footbridges, it is necessary to gather knowledge on their structural behaviour, particularly under dynamic loads, and to evaluate the ability of current design tools to predict their response. In fact, excessive vibration has a major influence on the in-service performance (comfort) of slender structures as well on their service life. The use of composite materials that combine high damping capacity with relatively high stiffness and low mass can provide functional and economic benefits, especially for footbridges. This paper aims to investigate the dynamic behaviour of GFRP free-supported beams to evaluate their modal characteristics (frequency, damping, and modal shape). To assess the benefits of using a structure made of pultruded GFRP rather than a conventional material—steel, a comparative analysis between the dynamic characteristics of GFRP and steel beams is performed. To specifically address material damping and to minimize the interference of the boundary conditions, the beams are tested in a free condition, resting on a low-density foam base. The results show that the damping capacity of GFRP is much higher than that of steel, as the measured damping factor of GFRP is five times higher than that of steel for the same boundary conditions and similar geometry. Furthermore, the fact that the frequencies of the tested specimens resemble for the two different materials highlights the perceived damping qualities of the polymer-based composite material. Finally, an energy method for evaluating the influence of the scale factor on material damping is applied, which made it possible to infer that the damping varies as a function of frequency but is not explicitly affected by the length of the specimens.

## 1. Introduction

Pultruded glass fibre reinforced polymer (GFRP) profiles are being increasingly considered for civil engineering structural applications, as a replacement of conventional materials, such as reinforced concrete and especially steel. This is mostly due to their lightweight, high strength, and non-corrodibility. Among the various applications of GFRP profiles are building structures and bridges, both vehicular and especially pedestrian. In all these applications, one of the main design requirements is the comfort of users and, for that purpose, the dynamic properties of fibre-polymer composite materials are of paramount importance. In the specific case of pedestrian bridges, design is very often governed by vibration criteria.

To allow for a comprehensive design of pedestrian bridges made of pultruded GFRP profiles, it is necessary to obtain an in-depth understanding of their dynamic properties, namely compared to steel, which is the main alternative material it aims at replacing. The high damping capacity of polymer-based materials is widely known and used in the mechanical, automotive, and aerospace industries. In fact, the vibrations produced in structures and mechanical components are the main sources of problems in high precision instruments, machines, automobiles or aircrafts, and the use of polymeric composites as passive damping elements allows mitigating vibrations in such structures [1,2]. The frequency-dependent stiffness behaviour of a viscoelastic material directly affects the modal characteristics of the structural system, resulting in complex vibration modes and differences in the relative phase of vibration [3].

In what concerns the use of material damping as the sole damping source of a structural system, Vasques et al. [4] pointed out that the viscoelastic damping has been used mainly as distributed surface mounted or embedded damping treatments, utilizing passive viscoelastic materials alone. In the same manner, Nashif et al. [5] introduced fundamentals of both vibrations and shock damping, focusing only on passive treatments for vibration attenuation without the effective use of the structure itself as a damping agent. Finegan and Gibson [6] summarised the research done on the damping of polymer-based composites in two levels: (a) At the macromechanical level, the research efforts aimed to study the properties of the laminate layers, the inertial effects and the contact surfaces; (b) at the micromechanical level, the focus has been on the effects of the orientation and ratio of the reinforcing fibres, the fibre/matrix interface and the properties of the fibres and the matrix. In the same study, the authors state that material damping can contribute to passive vibration control using the inherent ability of polymeric materials to dissipate energy. This ability was explored by Adams and Bacon [7] who examined the effect of fibre orientation and lamination geometry on the flexural and torsional damping and dynamic moduli of fibre reinforced polymer (FRP) composites.

As mentioned above, polymer-based composites are now finding increasing interest in civil engineering to be used as structural members (pultruded profiles, laminated shells and plates, and sandwich panels). Nevertheless, most of the research carried out in this area has focused on the evaluation of the static behaviour of composite structures, and less often on the dynamic behaviour of specific structures [8,9]; comprehensive information about the dynamic characteristics of polymer-based composites is not yet available, namely with respect to the material damping that is necessary to conduct dynamic analysis (in the time domain) under pedestrian loads, which are sometimes necessary to assess/design in a comprehensive way the comfort of users. This lack of information is well reflected in available design standards for composite structures, which provide very limited guidance in this respect (ASCE LFRD Standard [10]; Prospect for New Guidance of FRP Structures [11]; Italian Code [12]). In fact, none of the above-mentioned design guidelines give detailed information on material damping to be used in the dynamic analysis and design of fibre-polymer composite structures.

In this respect, Boscato et al. [13] stated that structures composed of pultruded FRP profiles have been extensively addressed in their static aspects, but the same attention has not yet been given to their dynamic behaviour. Regarding the dynamic behaviour of FRP materials, some studies have been conducted in the last two decades, such as that of Boscato et al. [14], who presented a modal identification of an all-FRP two dimensional frame in free vibration. The results show that the GFRP structural system presents a better dynamic performance compared to systems comprising steel and aluminium members. Stankiewicz et al. [15] presented dynamic in situ tests of a cable-stayed all-GFRP footbridge under human excitation. They found out that the analysed footbridge fulfilled the vibration comfort criteria elaborated by the technical guide Sétra [16].

In this paper, the identification of modal characteristics of free-supported beams is carried out to determine the internal damping of two types of materials: (i) Steel and (ii) a polymer-based composite. With this purpose, forced vibration tests using hammer excitation are conducted on two sets of similar free-supported beams, considering the lengths of 250, 500, and 1000 mm. A free-supported beam scenario, in which the beams are placed on a low-density polyurethane foam, is considered to avoid damping added by external sources and to minimise the interference from boundary conditions. In this way, it is possible to extract only the material damping ratio. These types of support conditions are common (and well-established) in biomechanical (body in free fall), naval (submarines), and aerospace (aircraft in flight) engineering. The different lengths aimed at verifying the relevance of the structure scale. The application of identification algorithms to the collected data enables the identification of modal properties, namely damping ratios, which are further correlated with damping factors extracted using an energy-based method analytically deduced in this research.

The remainder of this paper is organized as follows: In Section 2, a brief review of the free-supported beam theory is presented first, and the technique employed to identify modal properties is discussed next. The experimental programme is described in Section 3 and the results obtained are presented in Section 4. In Section 5, a comparison is made between modal parameters identified in the tests and the ones analytically calculated. In Section 6, damping estimates obtained for the two materials, GFRP and steel, are compared. Section 7 summarises the main findings of this study.

## 2. Theoretical Background

### 2.1. Identification of Modal Properties

The identification of modal properties from an existing structure is a so-called inverse problem, which is formulated from the non-homogenous dynamic equilibrium equation, by the relation between a response and an excitation, expressed either in the time or the frequency domain [17]. In the present case, a frequency-based approach is used and frequency response functions (FRF), $H\left(i\omega \right)$, are constructed from the ratio between spectral density functions of the applied modal force and the resulting modal response. From the FRF, the modal parameters are extracted, namely the natural frequencies, damping ratios, and mode shape components.

**Figure 1:**
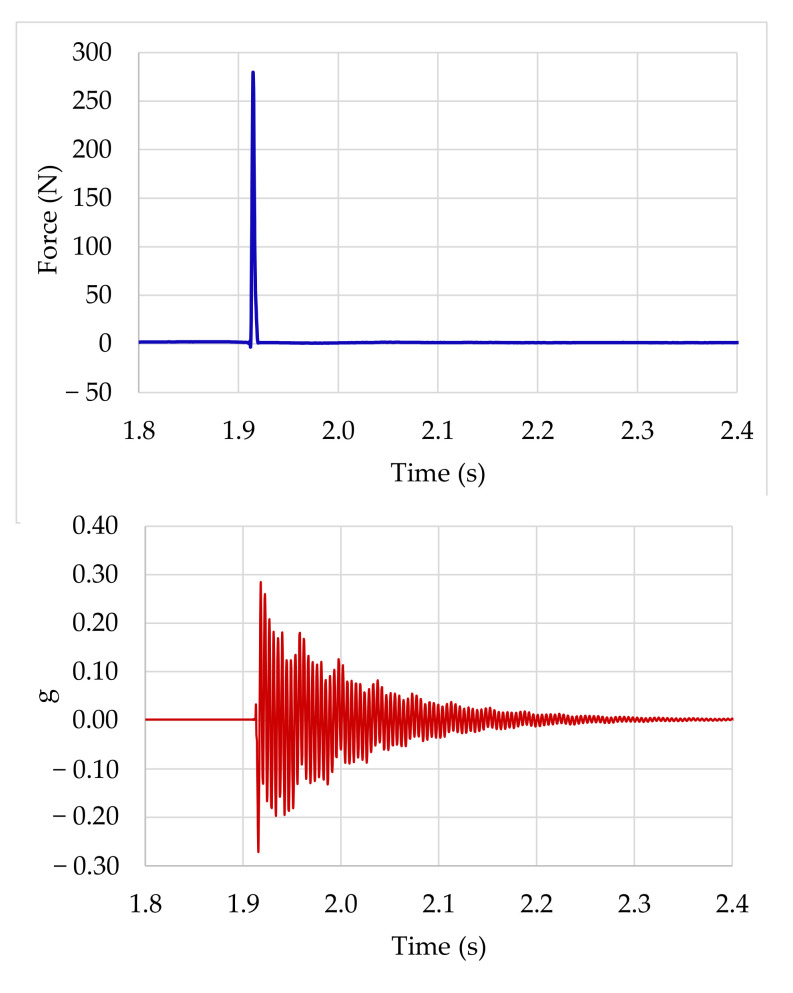
Input-output graphic from an impact experiment.

Figure 1 shows a typical input-output graphic of an experiment using an impact hammer excitation, depicting the typical response of a structural system to an imposed force.

In a system with separated natural frequencies, modal decoupling enables the transformation of the *N*-coupled dynamic equilibrium equations into *N*-decoupled single-degree-of-freedom (SDOF) equations, *N* being the number of vibration modes to identify. In the frequency domain, the general SDOF dynamic equilibrium equation is given by [18]

(1) $$X\left(\omega \right)\left(-\omega^{2}+2i\xi_{n}\omega \omega_{n}+\omega_{n}{}^{2}\right)=\frac{Y\left(\omega \right)}{m_{n}}$$

where $m_{n},\;\omega_{n}$, and $\xi_{n}$ are the modal mass, the circular frequency, and the damping ratio, respectively, i is the complex number $\left(i^{2}=-1\right)$, and $X\left(\omega \right)\;\mathrm{and}\;Y\left(\omega \right)$ are the Fourier transforms of the response and excitation, respectively. The FRF is defined as

(2) $$H\left(\omega \right)=\frac{X\left(\omega \right)}{Y\left(\omega \right)}$$

Equation (1) can then be expressed as

(3) $$H\left(\omega \right)=\frac{1/m_{n}}{\omega_{n}{}^{2}-\omega^{2}+i2\omega \omega_{n}\xi_{n}}$$

**Figure 2:**
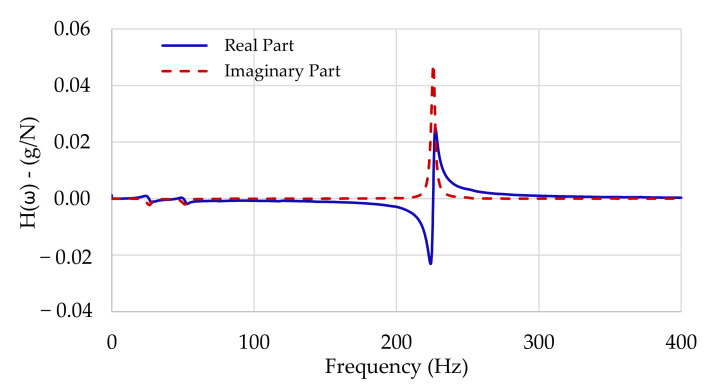
FRF in terms of real and imaginary parts.

Since the FRF is a complex function of frequency, the corresponding real and imaginary parts can be plotted, as shown in Figure 2. Accordingly, it can be observed that the imaginary part of the FRF presents peaks close to the resonance frequencies, while the real part inverts its signal in the vicinity of those frequencies.

These properties can be used to extract the relevant modal parameters. According to Rao [19], when using a single-degree-of-freedom approach, the graph of $H\left(i\omega \right)$ is divided into several frequency ranges, each one centred at one peak, whose abscissa approximately coincides with a resonance frequency for a lowly damped system.

To calculate the damping ratio $\xi_{n}$, the well-known half-power bandwidth method is commonly used [18]. This method consists of estimating $\omega_{1,n}$ and $\omega_{2,n}$ frequencies, around $\omega_{n}$, for which the respective FRF amplitude is equal to the peak divided by $\sqrt{2}$. Thereby, the modal damping ratio can be found by

(4) $$\xi_{n}=\frac{\omega_{2,n}-\omega_{1,n}}{2\omega_{n}}$$

**Figure 3:**
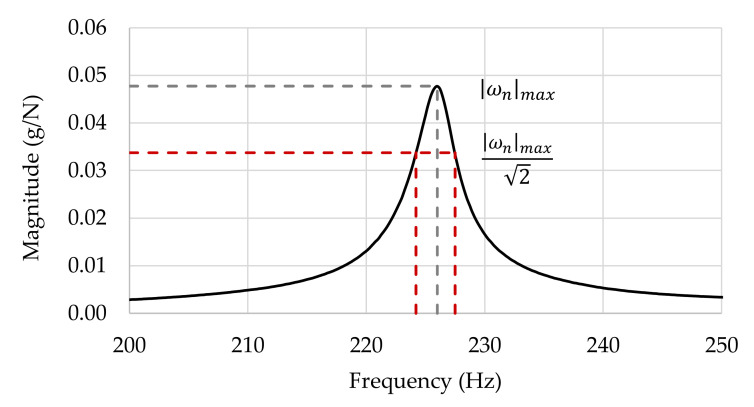
The logarithmic spectra of Fourier in amplitude.

Figure 3 shows the amplitude of the FRF in terms of logarithmic coordinates and exemplifies the application of the half-power bandwidth method.

The magnitude of the FRF is given by

(5) $$\bar{H_{ij}}\left(\omega_{n}\right)=\sqrt{{\left[H_{r,n}\left(\omega_{n}\right)\right]}^{2}+{\left[H_{i,n}\left(\omega_{n}\right)\right]}^{2}}$$

where $H_{r,n}$ and $H_{i,n}$ are the corresponding real and imaginary part, respectively.

Based on the $\omega_{n}$ and $\xi_{n}$ values, it is possible to estimate the amplitudes values, $\phi_{i,n}$ and $\phi_{j,n}$, at two measurement points, i and j, of the configuration relative to the mode n. As described by Caetano [18], for a given FRF, the values of $\phi_{i,n}$ and $\phi_{j,n}$, associated with a particular mode n, can be determined at the resonance for each mode. If $\omega ≅\omega_{n}$, then the amplitude of the FRF relating the response measured at point i with the force applied at point j is given by

(6) $$\bar{H_{ij}}\left(\omega_{n}\right)=\frac{\phi_{i,n}\cdot \phi_{j,n}}{2\xi_{n}\omega_{n}{}^{2}}$$

The amplitudes of the modal components are then given by

(7) $$\phi_{i,n}\cdot \phi_{j,n}=\bar{H_{ij}}\left(\omega_{n}\right)\cdot 2\xi_{n}\omega_{n}{}^{2}$$

The determination of the quantities $\phi_{i,n}$ and $\phi_{j,n}$ can follow a sequence. For example, if i=j, it follows that

(8) $$\phi_{i,n}=\sqrt{\bar{H_{ij}}\left(\omega_{n}\right)\cdot 2\xi_{n}\omega_{n}{}^{2}}$$

Then, at another point j

(9) $$\phi_{j,n}=\frac{\bar{H_{ij}}\left(\omega_{n}\right)\cdot 2\xi_{n}\omega_{n}{}^{2}}{\phi_{i,n}}$$

In summary, the FRF can be used to characterize the dynamic behaviour of a structure and contains information on the modal components of the system, namely the resonance frequencies, the damping ratios, and the mode shapes.

### 2.2. Energy-Based Evaluation of Equivalent Material Damping Ratio

As earlier related, the knowledge of the damping properties of a structure is necessary to effectively characterise its resonant response. In a structural system as a single-length beam, the energy dissipated per cycle of vibration can be attributed both to material damping and to damping in the supports [20].

Focusing specifically on material damping, the application of the energy method constitutes an alternative for determination of an equivalent damping ratio corresponding to each vibration mode n of the structure. This is achieved considering that the damping factor is given from the ratio between the dissipated energy, $W_{diss,n}$, and the total energy at resonance, $W_{total,n}$, over a vibration cycle [21]

(10) $$\xi_{n}=\frac{W_{diss,n}}{4\pi \;W_{total,n}}$$

For a single degree of freedom (SDOF) system, at resonance, the total energy may be calculated as the amplitude of either the maximum kinetic and potential energy [22]. If the kinematic energy is considered

(11) $$W_{total,n}={\left|\frac{1}{2}\rho A\int_{0}^{L}{\left(\frac{\partial y}{\partial t}\right)}^{2}dx\right|}_{t}$$

where y describes the transverse deflection of the beam at some position x, ρ is the volumetric mass, A is the cross-sectional area of the beam, the assumed constant along the length L, and ∂/∂t represents the partial derivative with respect to time.

In a harmonic vibration cycle, the maximum value of Equation (11) is given by

(12) $$W_{total,n}=\frac{1}{2}\left(L\rho A\right)\;\omega_{n}{}^{2}Y_{n}{}^{2}$$

where $Y_{n}$ is the displacement amplitude; k is the elastic stiffness; and LρA is the total mass.

Equations (12) and (13) shows that the energy of a harmonic oscillator is proportional to the square of the amplitude of the oscillation.

On another hand, the free vibrations of any real physical system decay with time. Every such system inevitably has dissipative features through which the mechanical energy of the vibration is depleted. In this sense, French [23] and King [24] showed that it is possible to express the damping ratio in terms of an exponential decay of the total mechanical energy, $W_{total,n}$.

In such conditions, the oscillations are well described over several cycles by a simple harmonic motion of constant amplitude Y, such that

(13) $$Y\left(t\right)=Y_{0}e^{-ht/2}$$

where $Y_{0}$ is the initial value of the amplitude and h is the hysteretic damping coefficient.

Hence, from Equation (13), one may define the decay of the total energy as

(14) $$W\left(t\right)=W_{total,n}\;e^{-ht}$$

Since the dissipated energy may be computed as $W_{total,n}-W\left(t\right)$, when $t=2\pi /\omega_{n}$, and replacing h by a constant given by λ, the damping ratio can be related to the energy decay as

(15) ξn=1−e−2πλωn4π

## 3. Experimental Programme

### 3.1. Overview

In this research, the identification of modal characteristics of free-supported beams is carried out to determine the structural damping of two types of materials, namely: (i) Steel and (ii) pultruded GFRP.

The experiments are conducted for different beam lengths, namely, 250, 500, and 1000 mm. The range of lengths chosen makes it possible to assess the scale effects on the modal properties of the beams, namely on their natural frequencies and damping ratios.

### 3.2. Characteristics of the Material and Specimens

#### 3.2.1. GFRP Specimens

**Figure 4:**
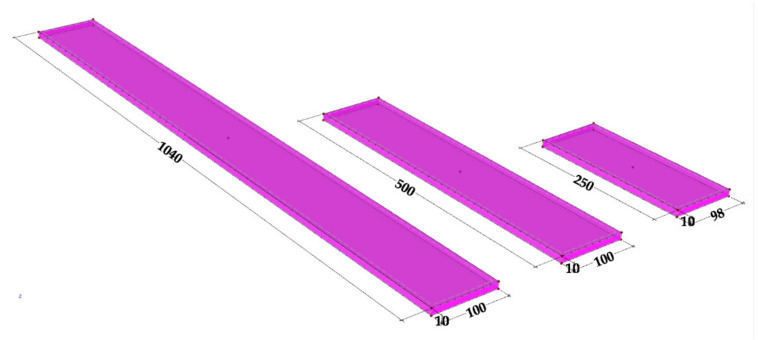
Geometry of GFRP specimens (dimensions in mm).

The specimens of the pultruded GFRP composite used in the experimental programme were extracted from the web of an I-section pultruded profile (200 × 100 × 10 mm, web height × flange width × wall thickness) (Alto-Perfis Pultrudidos Lda, Maia, Portugal). This profile is made of E-glass fibres, combining alternating layers of unidirectional roving and mats embedded in an isophthalic polyester matrix (68% inorganic content by weight). The cross-section and dimensions of GFRP specimens are shown in Figure 4 and their mass and density are shown in Table 1.

The dimensions of the composite specimens present some differences compared to the steel ones, which resulted from the cutting process. However, the relative differences are small and exact (measured) geometric values were considered in the calculations. Given the very low porosity of the pultruded material, the apparent density of the specimens was considered, by determining the weight/volume ratio.

The material characterisation tests [25] of GFRP were performed on small-scale coupons extracted from the web plate: (i) Tensile tests (according to ISO 527 [26]), (ii) compression tests (ASTM D695-02 [27]), (iii) in-plane shear tests (ISO 527-5 [28]), and (iv) interlaminar shear tests (ASTM D2344 [29]).

The elastic and strength properties of the GFRP laminates are listed in Table 2 and Table 3, respectively, where E is the elastic modulus, G is the shear modulus, υ is the Poisson ratio, σ is the axial strength, and τ is the in-plane shear strength. The subscripts L and T correspond to the in-plane longitudinal (pultrusion) and transverse directions, while subscripts t and c correspond to tensile and compressive loading, respectively.

#### 3.2.2. Steel Specimens

**Figure 5:**
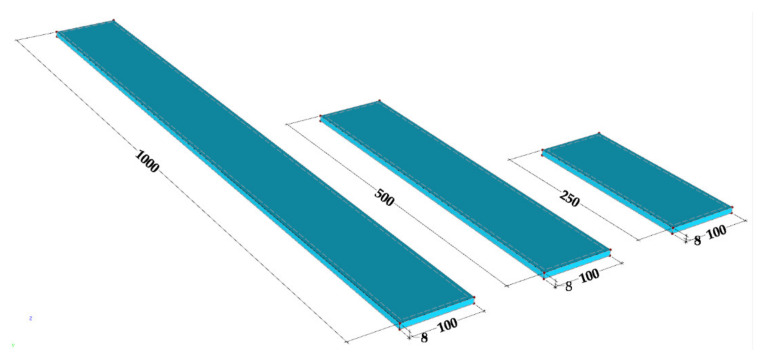
Geometry of steel specimens (dimensions in mm).

The steel specimens used in the experimental programme were produced with DIN 45WCrV7 steel grade, equivalent to ASTM 681A S1 or S355, a cold work alloy tool steel category. The cross-section and dimensions of steel specimens are shown in Figure 5 and their mass and density are listed in Table 4.

The elastic and strength properties of steel are listed in Table 5, namely the modulus of elasticity E, the minimum yield stress $f_{y}$, the ultimate tensile stress $f_{u}$, the shear modulus G, and the Poisson ratio υ (properties stated in the supplier catalogue).

### 3.3. Test Setup, Instrumentation, and Procedure

The modal identification tests were performed to extract the modal data from the specimens, namely, the vibration frequencies, damping ratios, and modal shapes of the beams. For this purpose, input-output tests were conducted based on the excitation by an impact hammer and the measurement of both the applied excitation and the structural acceleration. From the collected time series relating the acceleration at a certain number of points with the force applied at another point, frequency response functions were constructed, and modal identification techniques (described above) were applied.

**Figure 6:**
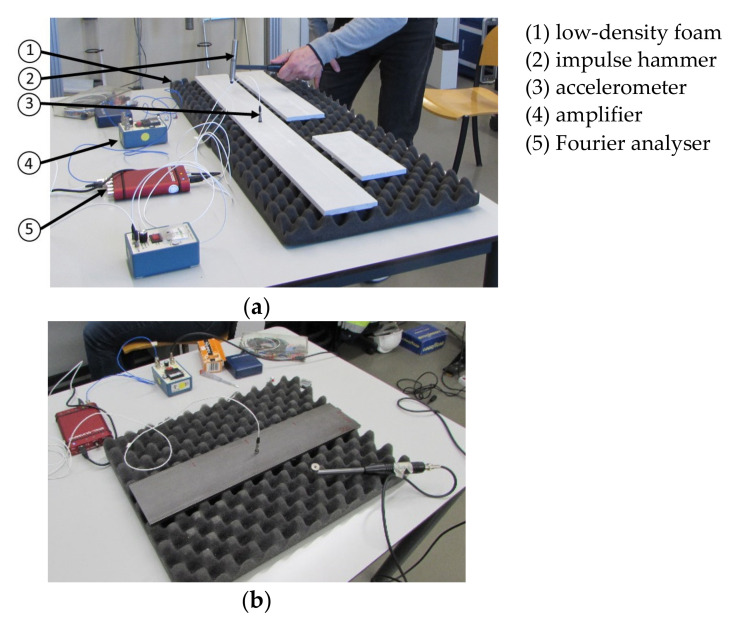
Experimental setup: (**a**) GFRP and (**b**) steel specimens.

Figure 6 shows the experimental setup. The test specimens were supported by a (1) low-density foam to simulate a free-supported condition. Impacts were applied with an (2) impulse hammer on the beam and the respective response was measured in terms of vertical accelerations with (3) a high sensitivity and low mass piezoelectric accelerometer (model 393A03, PCB Group, Inc., New York, NY, USA) connected to (4) an amplifier (model SignalCalc Ace, Data Physics, Santa Clara, CA, USA). The data were acquired and processed using a (5) Fourier analyser (model 480E09, PCB Group, Inc., New York, NY, USA).

The frequency range that can be induced by hammer excitation depends on the mass of the hammer and the hardness of the tip applied to its head. The chosen tip that allowed excitation in the frequency range of 0 to 800 Hz was a rubber- and steel-head for respectively the GFRP and steel specimens.

**Figure 7:**
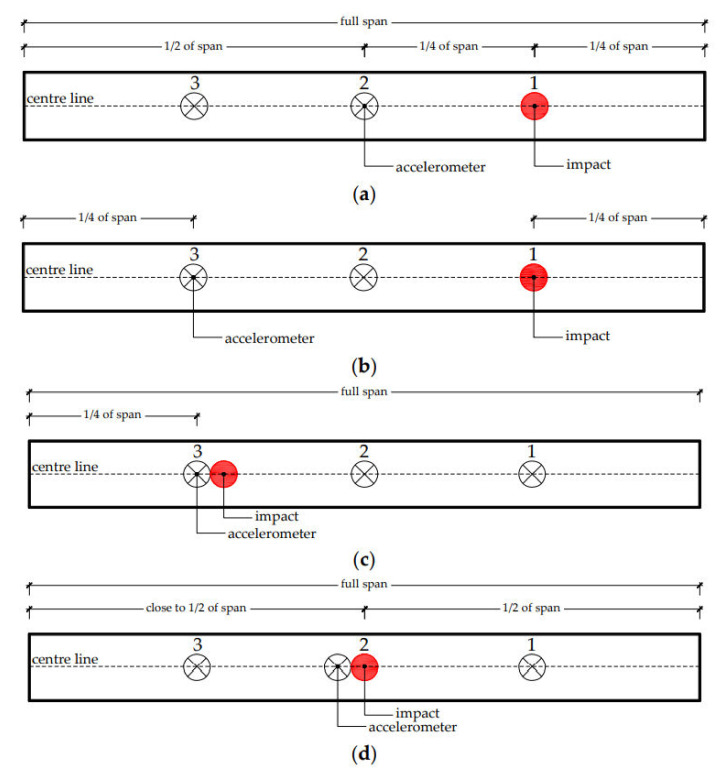
Modal identification test setups: (**a**) Setup 1, (**b**) setup 2, (**c**) setup 3, and (**d**) setup 4.

For each test conducted on, a given specimen (made of steel or GFRP), four different FRFs were constructed relating the accelerations measured at points 1, 2, and 3, with the impact applied at point 1, 2, and 3 (see Figure 7 and Table 6).

**Figure 8:**
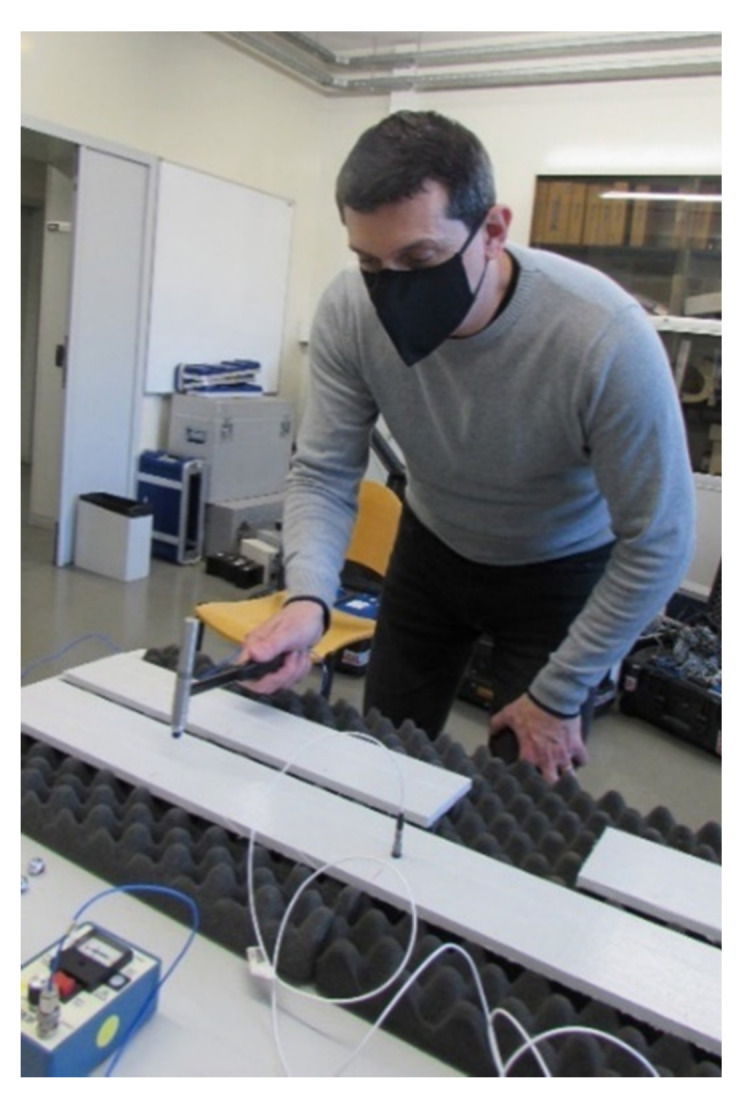
Experimental tests: Example of impact being applied.

The modal identification test setups described in Figure 7 were repeated for the three beam lengths. Figure 8 shows one of the modal identification tests being performed.

**Figure 9:**
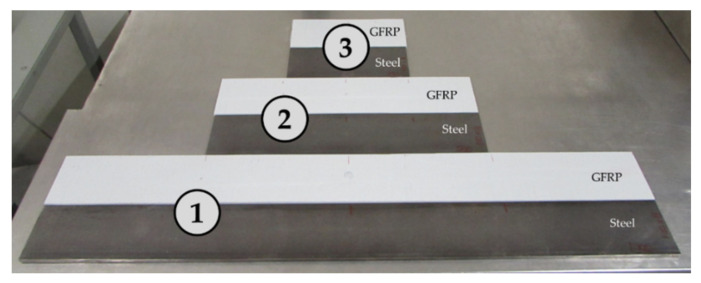
Experimental couples.

In this campaign, 19 tests were conducted and the 14 most relevant results are presented. Figure 9 shows the tested beams arranged in pairs, namely (1) 1000, (2) 500, and (3) 250 mm-length.

The series of experiments were classified following the setup number, as described above, the beam material (C—for composite; S—for steel) and the length (in mm). Table 7 describes the complete set of experiments performed for each series and the beam nomenclature adopted.

## 4. Experimental Results

This section describes the experimental results of the tests carried out on each pair of beams, made of either GFRP or steel. First, results are presented for each of the test lengths. Next, the identified frequencies are summarized. Finally, the mode shapes are described. 

### 4.1. 1000 mm-Length

**Figure 10:**
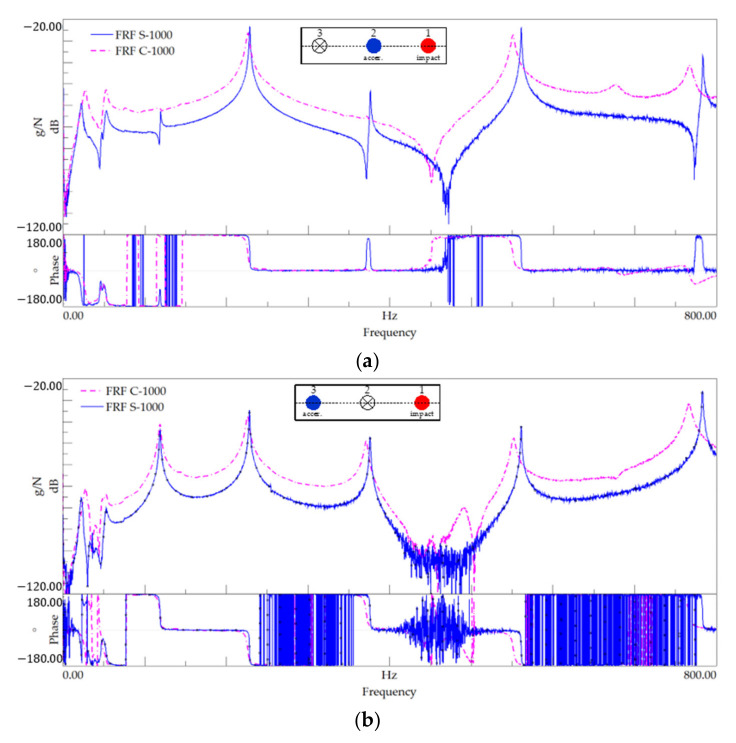

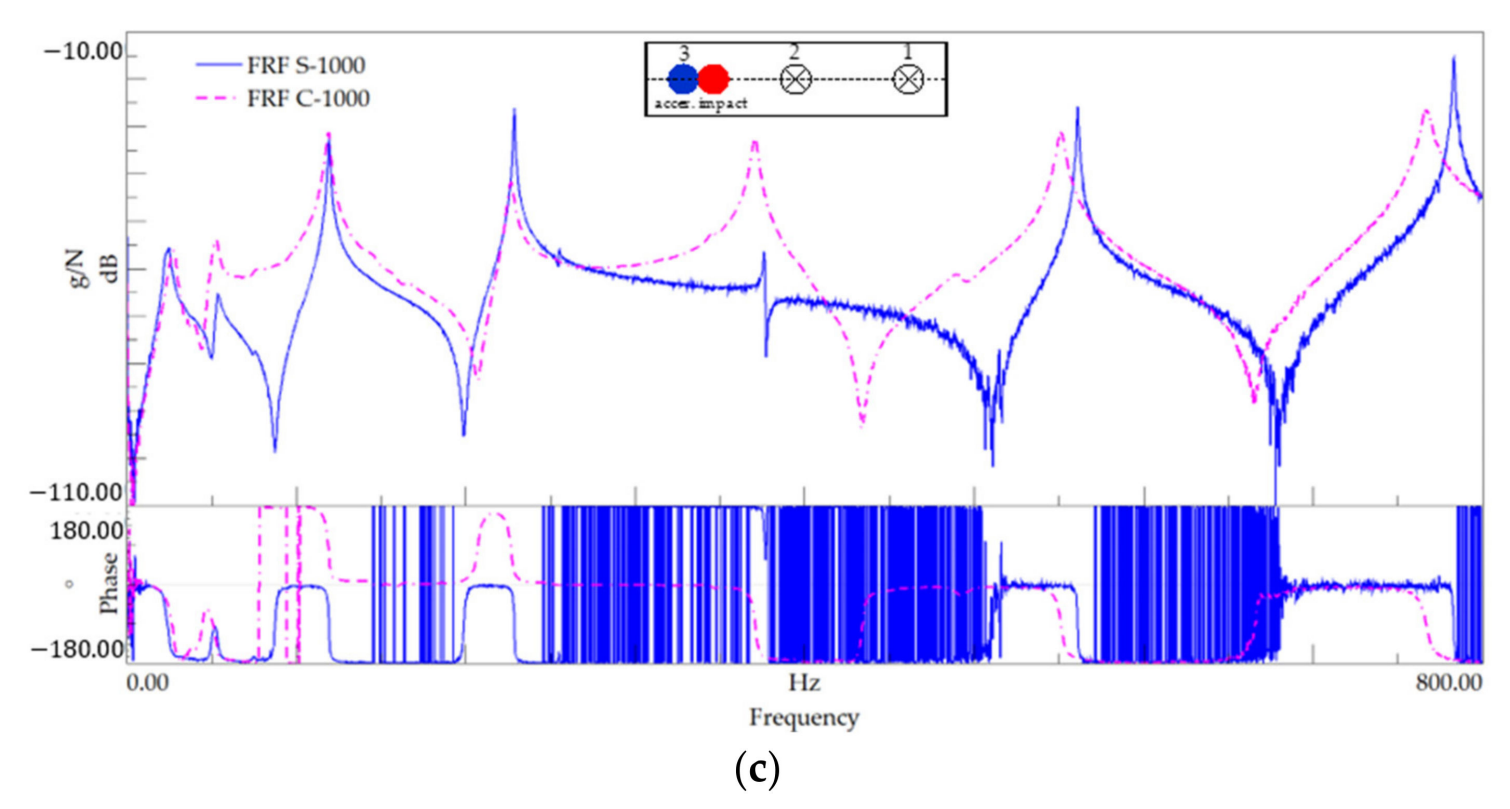
Modal identification for 1000 mm-length specimens—FRF plot for (**a**) setup 1, (**b**) setup 2, and (**c**) setup 3.

**Figure 11:**
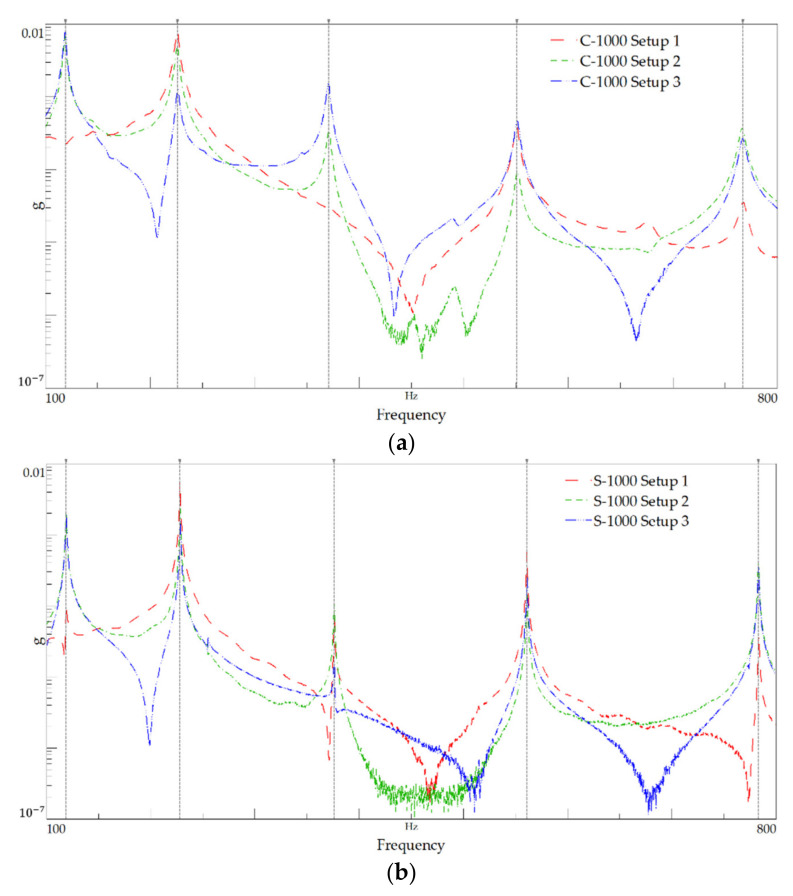
Power spectral density plot for the 1000 mm-length: (**a**) C-1000 and (**b**) S-1000.

This subsection presents the results of the modal identification test performed on specimens with 1000 mm of length, namely, for setups 1, 2, and 3 (Table 7). Figure 10 shows the FRF plots and summarises the identified natural frequencies. Figure 11 shows the power spectral density (PSD) of amplitude response plots of the 1000 mm-length tests. 

It is first noted that the plots presented in Figure 10 show that the resonance frequencies for both materials are very close. This is confirmed by the values of the resonance frequencies listed in Table 8.

### 4.2. 500 mm-Length

**Figure 12:**
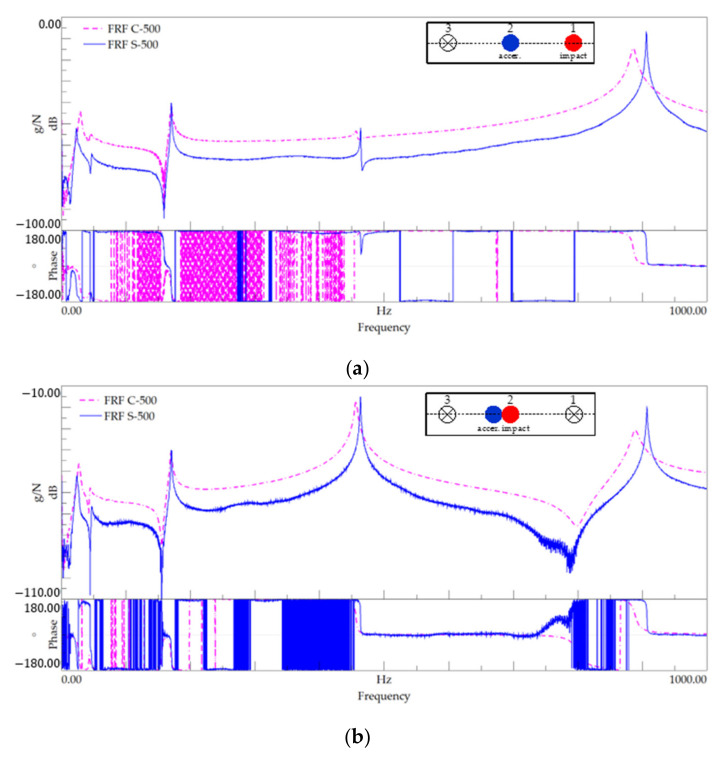
Modal identification for the 500 mm-length—FRF plot for (**a**) setup 1 and (**b**) setup 4.

**Figure 13:**
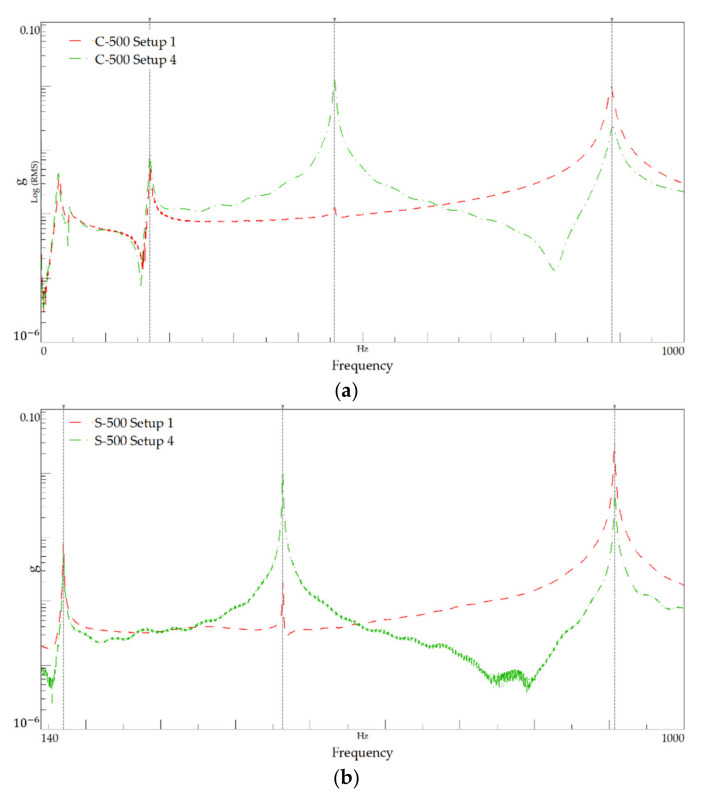
Power spectral density plot for the 500 mm-length: (**a**) C-500 and (**b**) S-500.

Figure 12 presents the FRF plots for setups 1 and 4, corresponding to modal identification tests performed on specimens with 500 mm-length, and Table 9 summarises the identified natural frequencies. Figure 13 shows the PSD of amplitude response plots of the 500 mm-length tests.

As in the previous series, it can be noted that the curves shown in Figure 12 and the values given in Table 9 show that the peak frequencies for both materials are close.

### 4.3. 250 mm-Length

**Figure 14:**
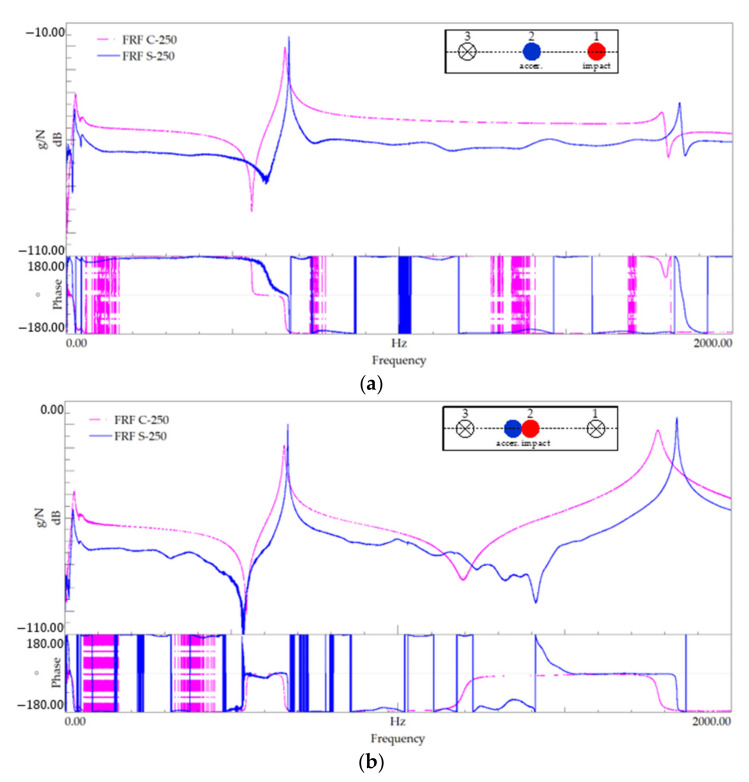
Modal identification for the 250 mm-length—FRF plot for (**a**) series 1 and (**b**) series 4.

**Figure 15:**
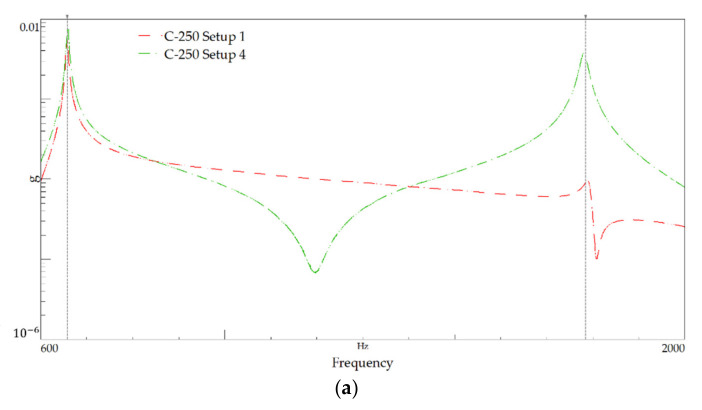

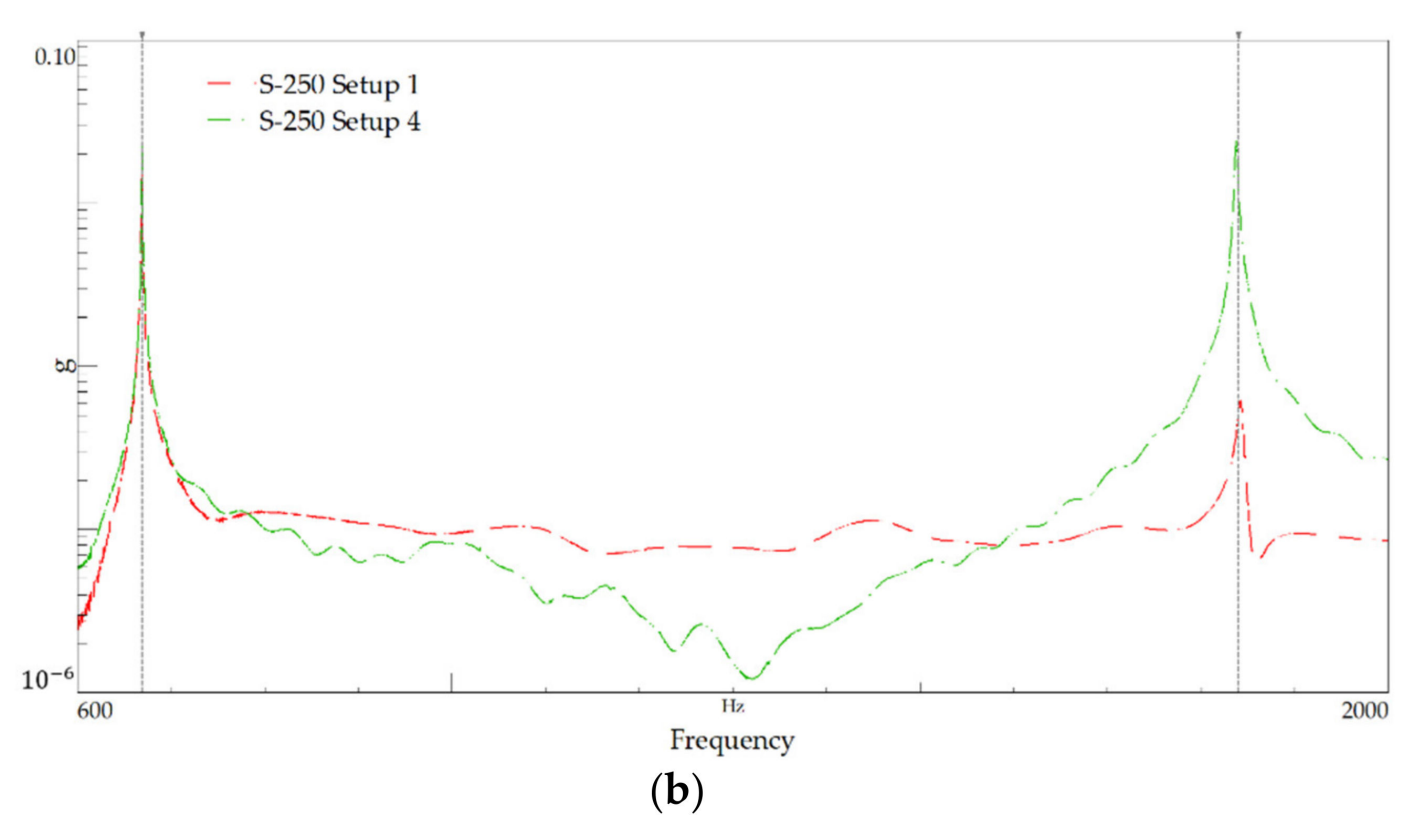
Power spectral density plot for the 250 mm-length: (**a**) C-250 and (**b**) S-250.

Figure 14 shows the FRF plots for setups 1 and 3, referring to modal identification tests performed on specimens with the 250 mm-length, and Table 10 summarises the identified natural frequencies. Figure 15 shows the PSD of amplitude response plots of the 250 mm-length tests.

**Figure 16:**
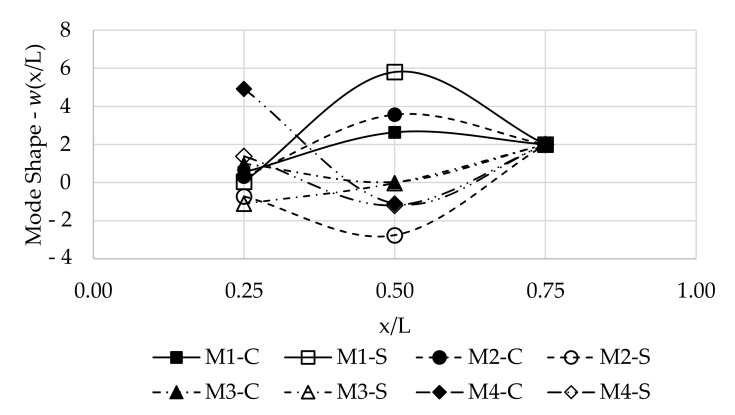
Modal identification: First four mode shapes with impact excitation. The prefix “M*i*” indicates the mode number, the suffix “-C” and “-S” indicates the material, composite (GFRP) and steel, respectively.

As mentioned in the previous tests, even for the small length beams, the peak frequencies are very close for both materials, as depicted by the FRF plots in Figure 16 and the values listed in Table 10.

### 4.4. Summary of Identified Frequencies

Table 11 summarises the identified frequencies for the different materials-lengths and modes. The frequencies associated with the mode identified as “0” correspond to the rigid-body mode and are discarded. From the values shown in Table 11, it is possible to verify the existence of similarity in the vibratory behaviour of beams with the same length, but different constituent materials. To confirm this hypothesis, Table 12 shows the ratio between the identified frequencies for GFRP and steel beams with the same length, for the same vibration mode.

The values listed in Table 12 show that the relative difference between the values of the identified frequencies for both materials remains within the range of 0 to 0.03, which reflects the resemblance of modal characteristics of specimens with the same length, regardless of the material. It is important to note that, for the same length, while the bending stiffness ratio is about three times (the estimated bending stiffness of the steel specimens being greater than that of the composite ones), the stiffness-to-mass ratio (composite/steel) of those specimens is less than 8% on average, as already mentioned. This is ascribed to the much lower density of GFRP compared to steel.

### 4.5. Mode Shapes

To obtain the solution of the vibration modes, the Euler-Bernoulli theory [30] was applied and the modal displacement amplitudes of the beams were calculated. The first four mode shapes are presented in Figure 16, in which the prefix “M*i*” indicates the mode number, and the suffix “-C” and “-S” indicates the material, composite (GFRP) and steel, respectively. It is noted that it was not the purpose of the tests to define in a refined way the modal shapes, as a small number of points were used in the measurements.

## 5. Comparison of Identified and Calculated Modal Parameters

This section presents a comparison between the modal parameters obtained from the analytical formulae and the experimental results. For this purpose, the Euler-Bernoulli theory [30] and the values of the corresponding wavenumber are used.

Table 13 summarizes the elastic properties of the beams in which the “Ratio EI/m” column emphasizes the relationship between the bending stiffness and the mass of the specimen. It is possible to verify that the relationship “stiffness × mass” for specimens of the same geometry is similar, with a relative difference of only 8% between both (with the calculated ratio for the GFRP composite being higher than that for steel). Table 14 shows the analytical values of natural frequencies, $f_{n}$, given by Equation (9).

According to the Euler-Bernoulli theory, the natural frequency is inversely proportional to the square of specimen length. In this sense, as the EI/m ratios are similar between specimens with the same geometry, it is expected that the natural frequencies of the specimen C-1000 mm are lower than those of the specimen S-1000, since the length of the latter is 3.85% smaller than that of the former, it did not occur with the other specimens.

A comparison between the analytical and experimental values of natural frequencies is made to assess the accuracy of the analytical modelling and the test approach, and further, to validate the accuracy of the proposed model in simulating the free-supported beam boundary conditions.

Table 15 summarises the ratios between the experimental and numerical values of the natural frequencies for the different materials-lengths and for each mode.

In what concerns the accuracy aspect mentioned above, a value of ±0.10 was defined as an acceptable threshold. In this sense, the first mode of specimens C-1000 and S-1000 should be discarded from the analysis. Except for the first mode of the 1000 mm-length specimens, the good agreement between the experimental frequencies and those estimated with the analytical models shows that the hypothesis of the free-supported condition was achieved with the use of low-density foam as beams supports. It was not possible to unequivocally identify a clear cause for the differences between experimental and analytical results.

## 6. Damping Analysis

In this section, the damping ratios extracted for each pair of specimens with an identical length is analysed and compared. The procedure described in Section 2 is applied to calculate the damping ratios.

From the power spectrum of amplitude response plots, the half-power bandwidth method is used and the damping ratio is calculated. The values obtained are shown in Table 16.

The values given in Table 17 show that for a given mode, the damping ratio, $\xi_{n}$, of the GFRP composite is higher than that of the corresponding steel-based specimen. It must be noted that for the same structure, at the same frequency, different response amplitudes will generate different levels of damping [31]. In this sense, it is important to assess the damping ratio concerning the ratio of the response amplitudes of the structure. 

To confirm the conclusion on the enhanced damping ability of the GFRP composite with respect to steel, the evaluation of the relative response amplitudes associated with each mode component for the two different materials is performed using the power spectrum amplitude response. Table 17 summarises the amplitude response for each identified mode and the ratio between the measured amplitudes (GFRP/steel) as well as the ratio between the measured damping (GFRP/steel).

Table 17 shows that the response amplitude for the various identified modes has an average ratio of 1.4 between the GFRP composite and steel specimens, while the average ratio of damping between those two materials is 4.0. These two figures highlight the fact that the difference in measured amplitudes does not have a direct impact on the damping values. It should be noted that at certain modes the response amplitude for steel was higher than in the GFRP composite.

**Figure 17:**
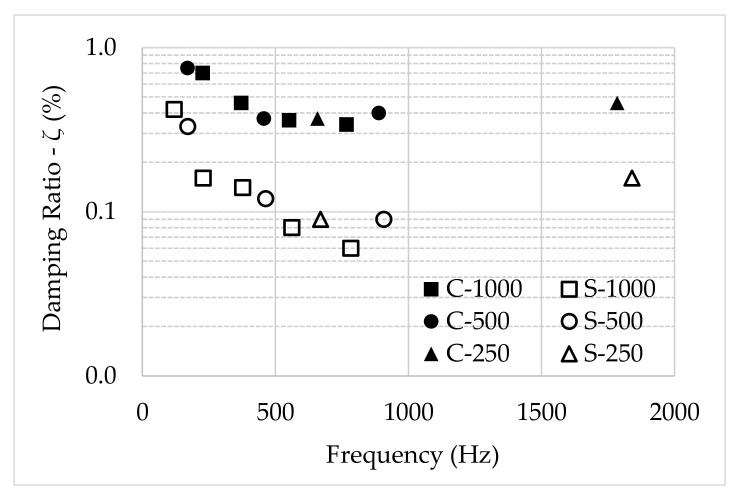
Damping ratio vs. frequency for different materials and lengths.

A consideration to be made refers to the scaling effect, which relates the length of the specimens to the damping variation, within the same frequency range, and which is directly related to the choice of specimen lengths adopted in this research. Furthermore, the natural frequencies of a full-scale system can be related to those of a reduced model, relating the two sets of frequencies by a scaling law taking into account the physical parameters of the model and the full-scale system [32]. In this sense, the damping ratio as a function of the length (scale) was evaluated to identify whether the scale influences material damping. Figure 17 shows the variation of the damping ratio with the frequency for all tested specimens. The damping ratio decreases as the frequency increases, up to a frequency of approximately 700 Hz. At high frequencies (above 800 Hz), the damping ratio starts to increase.

**Figure 18:**
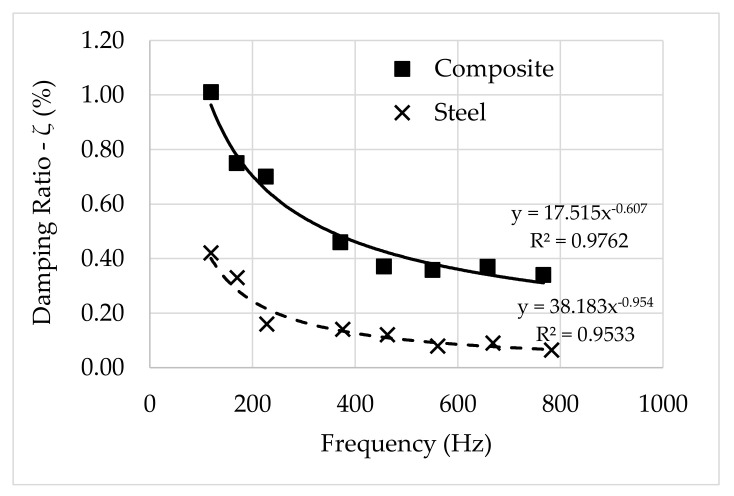
Damping ratio vs. frequency: Curve fitting.

It may be noted that the material damping varies as a function of frequency but is not explicitly affected by the length of the specimens. As far as only material damping is concerned, the damping ratio is similar for specimens with different lengths, at the same frequency range. This is corroborated by a curve fitting in power law terms that exhibits a very-strong correlation between the damping ratio values and frequency for different lengths, as depicted in Figure 18.

**Figure 19:**
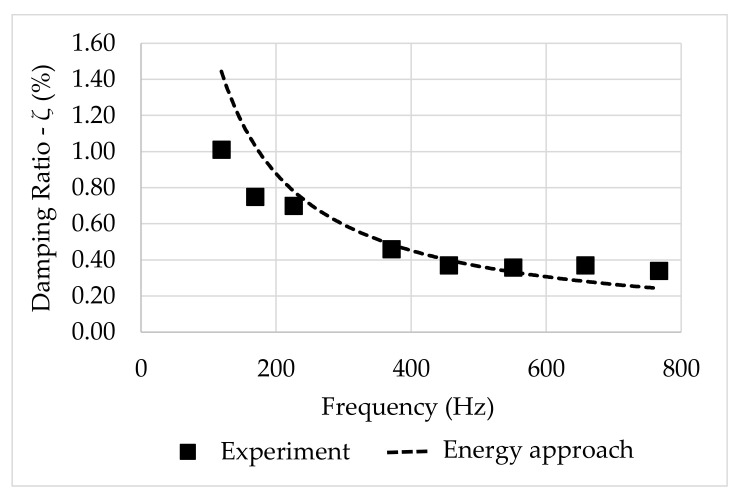
Damping ratio: Curve fitting from the energy method.

The curve fittings depicted in Figure 18 can be compared to those obtained based on the energy method. From Equation (15), one may evaluate the damping ratio as a function of a constant λ, in terms of exponential decay. In this sense, the results obtained for the GFRP composite, depicted in Figure 19, show that the damping ratios estimated based on the energy ratio are similar to the ones calculated from the curve fitting described above. 

## 7. Conclusions

This paper presents a modal analysis of the dynamic characteristics of free-supported beams, made of pultruded GFRP and steel, to identify and compare their resonance frequencies, damping ratios, and vibration modes.

For the modal identification process, an experimental campaign was conducted by applying the input-output method with the hammer impact and measuring the responses in terms of acceleration of specimens with various lengths. This approach allowed evaluating the damping variation as a function of resonance frequencies as well as achieving a wide range of frequencies, from 27 to 1800 Hz.

The comparison between the identified frequencies and the results obtained from the analytical model showed that the use of low-density foam as support enable a free-support beam condition, also allowing the retrieval of the material damping ratios from the test specimens. The same comparison showed a resemblance between the identified frequency for specimens with similar lengths but made of different materials. Furthermore, the order of magnitude of the relative differences between the frequencies identified for the same mode in the two different materials, did not exceed 3%.

The half-power bandwidth method was applied to the PSD plots and values of material damping ratios could be identified. For similar lengths, the values of the damping ratios of the two materials presented significant differences, with the GFRP composite presenting higher values, between two and six times higher than steel. This better damping behaviour of GFRP was confirmed through the evaluation and comparison of the measured acceleration amplitudes: The ratio of acceleration amplitudes between GFRP and steel was relatively small compared to the corresponding ratio of damping ratios. It is noteworthy that, for certain modes, the response amplitude of steel was higher than that of the GFRP. These results reflect the better performance of the polymer-based composite material in terms of energy dissipation and vibration attenuation.

Finally, an evaluation of the importance of the beam length was assessed to identify its influence on the material damping ratios. It was observed that the material damping ratio varies as a function of frequency but is not explicitly affected by the length of the specimens.

For the materials and geometries used in this study, it was shown that longer specimens exhibited modal characteristics that resemble those of the shorter specimens. Despite the limitations of this study, this points to the feasibility of estimating the material damping of full-scale structures based on small-scale tests at the material level.

## Figures and Tables

**Table 1:** Mass and density of GFRP specimens.

Specimen	Mass	$\mathrm{Volumetric}\;\mathrm{Mass}\;\left(\rho \right)$
(l × w × t)	(kg)	(kg/m^3^)
1040 × 100 × 10 mm	2.0088	1931.54
500 × 100 × 10 mm	0.9904	1980.80
250 × 98 × 10 mm	0.4891	1996.33

**Table 2:** Elastic properties of GFRP.

$E_{L,t}$	$E_{L,c}$	$E_{T,c}$	$G_{LT}$	υ
(GPa)	(GPa)	(GPa)	(GPa)	
32.7	33.4	10.8	3.65	0.266

**Table 3:** Strength properties of GFRP.

$\sigma_{L,t}$	$\sigma_{L,c}$	$\sigma_{T,c}$	$\tau_{LT}$
(MPa)	(MPa)	(MPa)	(MPa)
365	468	110	30.6

**Table 4:** Mass and density of steel specimens.

Specimen	Mass	$\mathrm{Volumetric}\;\mathrm{Mass}\;\left(\rho \right)$
(l × w × t)	(kg)	(kg/m^3^)
1000 × 100 × 8 mm	6.1611	7701.38
500 × 100 × 8 mm	3.0802	7700.50
250 × 100 × 8 mm	1.5417	7865.82

**Table 5:** Elastic and strength properties of A681 Steel.

E	$f_{y}$	$f_{u}$	G	υ
(GPa)	(MPa)	(MPa)	(GPa)	
210	335	510	80	0.300

**Table 6:** Measurement setting.

Setup	Chanel 1	Chanel 2
	Impact Load Point	Accelerometer Point
1	1	2
2	1	3
3	3	3
4	2	2

**Table 7:** Test description.

Test	Setup	Impact Load Point	Accelerometer Point	Beam Nomenclature
1	1	1	2	C-1000
2	2	1	3	C-1000
3	3	3	3	C-1000
4	1	1	2	S-1000
5	2	1	3	S-1000
6	3	3	3	S-1000
7	1	1	2	C-500
8	4	2	2	C-500
9	1	1	2	S-500
10	4	2	2	S-500
11	1	1	2	S-250
12	4	2	2	S-250
13	1	1	2	C-250
14	4	2	2	C-250

**Table 8:** Identified natural frequencies for the 1000 mm-length.

Mode	C-1000	S-1000
fNo.	Resonance Freq.	Resonance Freq.
	(Hz)	(Hz)
0	27.333 ± 0.312	23.000 ± 1.080
1	52.500 ± 0.204	53.127 ± 0.625
2	118.875 ± 0.125	119.083 ± 0.285
3	226.250 ± 0.205	228.417 ± 0.192
4	370.875 ± 0.375	376.083 ± 0.176
5	551.000 ± 0.204	561.083 ± 0.118
6	766.667 ± 0.514	783.334 ± 0.204

**Table 9:** Identified natural frequencies for the 500 mm-length.

Mode	C-500	S-500
No.	Resonance Freq.	Resonance Freq.
	(Hz)	(Hz)
0	27.656 ± 1.094	23.125 ± 0.313
1	44.219 ± 0.156	46.563 ± 0.001
2	169.219 ± 0.156	170.156 ± 0.156
3	456.406 ± 0.156	463.281 ± 0.156
4	887.504 ± 1.559	907.031 ± 0.469

**Table 10:** Identified natural frequencies for the 250 mm-length.

Mode	C-250	S-250
No.	Resonance Freq.	Resonance Freq.
	(Hz)	(Hz)
0	27.817 ± 0.004	24.375 ± 0.625
1	657.501 ± 0.624	668.594 ± 0.156
2	1784.362 ± 4.674	1840.000 ± 2.188

**Table 11:** Summary of identified frequencies (*in Hz*).

Specimen	Mode						
	0	1	2	3	4	5	6
C-1000	27.33	52.50	118.88	226.25	370.88	551.00	766.67
S-1000	27.00	53.13	119.08	228.42	376.08	561.08	783.33
C-500	44.22	169.22	456.41	887.50			
S-500	46.56	170.16	463.28	907.03			
C-250	27.82	657.50	1784.36				
S-250	24.38	668.59	1840.00				

**Table 12:** Ratio of identified frequencies for different setups.

Specimen	Mode					
	1	2	3	4	5	6
C-1000/S-1000	0.99	1.00	0.99	0.99	0.98	0.98
C-500/S-500	0.99	0.99	0.98			
C-250/S-250	0.98	0.97				

**Table 13:** Elastic properties set for the direct problem.

Specimen	Length	Cross-Section	Young’s Modulus	Moment of Inertia	Mass	Ratio
	*L*	*(w x t)*	*E*	*I*	*m*	*EI*/*m*
	(mm)	(mm)	(GPa)	(mm^4^)	(kg)	
C-1000	1040	100 × 10	37.7	8333.33	2.0088	156
S-1000	1000	100 × 8	210.0	4266.67	6.1611	145
C-500	500	100 × 10	37.7	8333.33	0.9904	317
S-500	500	100 × 8	210.0	4266.67	3.0802	291
C-250	250	98 × 10	37.7	8166.67	0.4891	630
S-250	250	100 × 8	210.0	4266.67	1.5417	581

**Table 14:** Natural frequencies obtained from analytical modelling (in Hz).

Specimen	Mode					
	1	2	3	4	5	6
C-1000	41.987	115.738	226.893	375.065	560.282	782.543
S-1000	42.941	118.369	232.051	383.592	573.020	800.334
C-500	179.378	494.463	969.345	1602.376	2393.674	3343.230
S-500	171.775	473.504	928.257	1534.456	2292.212	3201.519
C-250	714.717	1970.145	3862.272	6384.532	9537.389	13,320.816
S-250	686.743	1893.032	3711.100	6134.637	9164.089	12,799.430

**Table 15:** Ratios between experimental and analytical frequencies.

Specimen	Mode					
	1	2	3	4	5	6
C-1000	1.25	1.03	1.00	0.99	0.98	0.98
S-1000	1.24	1.01	0.98	0.98	0.98	0.98
C-500	0.94	0.92	0.92			
S-500	0.99	0.98	0.98			
C-250	0.92	0.91				
S-250	0.97	0.97				

**Table 16:** Damping ratio −$\xi_{n}$.

**Mode**	**C-1000**	**S-1000**	**Ratio**
	**(%)**		**(C/S)**
1	-	-	-
2	1.01	0.42	2.4
3	0.70	0.16	4.4
4	0.46	0.14	3.3
5	0.36	0.08	4.5
6	0.34	0.06	5.7
**Mode**	**C-500**	**S-500**	**Ratio**
	**(%)**		**(C/S)**
1	0.75	0.33	2.3
2	0.37	0.12	3.1
3	0.40	0.09	4.4
**Mode**	**C-250**	**S-250**	**Ratio**
	**(%)**		**(C/S)**
1	0.37	0.09	4.1
2	0.46	0.16	2.9

**Table 17:** Amplitudes of acceleration and the respective damping ratios.

**Mode**	**C-1000**		**S-1000**		**Ratios**	
	**Amplitude**	**Damping**	**Amplitude**	**Damping**	**Amplitude**	**Damping**
	**(g/N)**	**%**	**(g/N)**	**%**	**(C/S)**	**(C/S)**
1	-	-	-	-	-	-
2	0.0276	1.01	0.0236	0.42	1.2	2.4
3	0.0083	0.70	0.0493	0.16	0.2	4.4
4	0.0113	0.46	0.0015	0.14	7.8	3.3
5	0.0283	0.36	0.0516	0.08	0.6	4.5
6	0.0480	0.34	0.1700	0.06	0.3	5.7
**Mode**	**C-500**		**S-500**		**Ratios**	
	**Amplitude**	**Damping**	**Amplitude**	**Damping**	**Amplitude**	**Damping**
	**(g/N)**	**%**	**(g/N)**	**%**	**(C/S)**	**(C/S)**
1	0.0088	0.75	0.0097	0.33	0.9	2.3
2	0.1413	0.37	0.1723	0.12	0.8	3.1
3	0.1881	0.40	0.4178	0.09	0.5	4.5
**Mode**	**C-250**		**S-250**		**Ratios**	
	**Amplitude**	**Damping**	**Amplitude**	**Damping**	**Amplitude**	**Damping**
	**(g/N)**	**%**	**(g/N)**	**%**	**(C/S)**	**(C/S)**
1	0.0865	0.37	0.0844	0.09	1.0	4.1
2	0.0036	0.46	0.0051	0.16	0.7	2.9

## Data Availability

The data presented in this study are available upon request from the corresponding author.

## Nomenclature

**Symbol**	**Description**
$m_{n}$	modal mass of nth order mode (kg)
$\omega_{n}$	circular frequency of nth order mode (rad/s)
$\xi_{n}$	damping ratio of nth order mode (%)
i	complex number $\left(i^{2}=-1\right)$
$X\left(\omega \right)$	Fourier transform of the response
$Y\left(\omega \right)$	Fourier transforms of the excitation
$H\left(\omega \right)$	frequency response function (FRF)
$H_{r,n}$	real part of FRF at the circular frequency $\omega_{n}$
$H_{i,n}$	imaginary part of FRF at $\omega_{n}$
$\phi_{n}$	amplitude of vibration related to mode n
$W_{diss,n}$	dissipated energy at resonance in mode n
$W_{total,n}$	total energy at resonance in mode n
ρ	volumetric mass (kg/m3)
A	cross-sectional area (m2)
L	length of beam (m)
$Y_{n}$	modal displacement amplitude at nth order mode (m)
k	elastic stiffness (kN/m)
E	elastic modulus (GPa)
G	shear modulus (GPa)
υ	Poisson ratio
σ	axial stress (MPa)
τ	in-plane shear stress (MPa)
$f_{y}$	minimum yield stress (MPa)
$f_{u}$	tensile stress (MPa)
I	second moment of area of A (mm4)
